# Clinical Outcomes of Drug-Coated Balloon Treatment After Successful Revascularization of *de novo* Chronic Total Occlusions

**DOI:** 10.3389/fcvm.2022.821380

**Published:** 2022-04-13

**Authors:** Eun Jung Jun, Eun-Seok Shin, Eu-Vin Teoh, Youngjune Bhak, Song Lin Yuan, Chong-Mow Chu, Scot Garg, Houng Bang Liew

**Affiliations:** ^1^Department of Cardiology, Ulsan Medical Center, Ulsan, South Korea; ^2^Department of Cardiology, Ulsan University Hospital, University of Ulsan College of Medicine, Ulsan, South Korea; ^3^Cardiology Department and Clinical Research Center, Queen Elizabeth Hospital II, Kota Kinabalu, Malaysia; ^4^Department of Biomedical Engineering, College of Information-Bio Convergence Engineering, Ulsan National Institute of Science and Technology (UNIST), Ulsan, South Korea; ^5^Department of Cardiology, Dong-A University Hospital, Busan, South Korea; ^6^Department of Cardiology, East Lancashire Hospitals NHS Trust, Lancashire, United Kingdom

**Keywords:** *de novo*, clinical outcome, coronary artery disease, drug-coated balloon (DCB), chronic total occlusion (CTO)

## Abstract

**Background:**

The safety and efficacy of drug-coated balloon (DCB) treatment for *de novo* coronary chronic total occlusion (CTO) remain uncertain. The aim of this study was to evaluate the outcomes of DCB only treatment for *de novo* CTO.

**Methods:**

In this retrospective study, 101 vessels with *de novo* CTO lesions dilated by balloon angioplasty with thrombolysis in myocardial infarction flow grade 3 were included. Among them, 93 vessels successfully treated with DCB only treatment were analyzed. The study endpoint was major adverse cardiac events (MACE) at 2 years, a composite of cardiac death, non-fatal myocardial infarction (MI), target vessel revascularization (TVR), and target vessel thrombosis. The secondary endpoint was late lumen loss (LLL) on follow-up coronary angiography.

**Results:**

All 84 patients were followed up clinically, and 67 vessels underwent scheduled coronary angiography after 6 months. There were no procedural complications, and three vessels required bailout-stenting. The median follow-up was 720 days (interquartile range [IQR]; 406–1,268 days). MACE occurred in 8.3% of the patients after 1 year, including cardiac death (1.2%), TVR (7.1%), and no non-fatal MI and target vessel thrombosis. Two years after treatment, MACE occurred in 16.7% of the patients, including cardiac death (2.4%), non-fatal MI (3.6%), TVR (13.1%), and no target vessel thrombosis. The mean LLL was 0.03 ± 0.53 mm. Binary restenosis occurred in 14.9% of the treated vessels, and 3.0% of the vessels had late re-occlusion on follow-up coronary angiography.

**Conclusions:**

If the result of revascularization using balloon angioplasty is good, the clinical outcomes of DCB only treatment of *de novo* CTOs at the 2-year follow-up are encouraging, with a low rate of hard endpoints and acceptable MACE rates (Clinical Trial Registration Information; Impact of Drug-coated Balloon Treatment in *de novo* Coronary Lesion; NCT04619277).

## Introduction

Chronic total occlusions (CTOs) account for 20% of the lesions detected using coronary angiography, and approximately half of them are treated with revascularization, whereas the other half are treated with medical therapy (1). Recently, percutaneous coronary intervention (PCI) for CTOs has rapidly evolved, and although there have been major improvements in equipment and techniques, CTO remains one of the biggest challenges for interventional cardiologists, especially as the risks of restenosis and stent thrombosis (ST) remain high (2). This aspect is most problematic for vessels distal to the occlusion, which often enlarge after the restoration of antegrade flow; therefore, residual distal stenoses observed immediately after PCI of the CTO that do not affect antegrade flow may not require stenting (3).

There is much evidence that drug-coated balloon (DCB) treatment results in lower rates of restenosis and thrombosis and better long-term outcomes when used for PCI of in-stent restenosis (ISR) compared to plain old balloon angioplasty or additional stenting with drug-eluting stents (DES) (4–6). Considering the prevalence of CTOs and their high risk of restenosis and ST following PCI, investigating the safety and efficacy of DCB treatment in CTO PCI is necessary (7, 8).

Studies on DCB only treatment for CTO are scarce; however, recent registry data suggest that it is a feasible and well-tolerated treatment, provided that the result from pre-dilation is good (9, 10). In the feasibility and safety assessment, the incidence of angiographic restenosis was 11.8% at a mean follow-up of 8 months. This was not higher than prior results on CTO using either DES (14.2%) or bare-metal stents (36.6%) of 15 studies that included 3,193 patients (10). Furthermore, positive late lumen gain was reportedly found in 67.6% of patients, which was attributed to an increase in DCB-treated vessel size (9). Although registry data showed that DCB only treatment for CTO is feasible, the clinical impact of DCB treatment on CTO PCI remains uncertain. Therefore, this study aimed to evaluate the clinical outcomes of DCB only treatment approach without stenting for *de novo* coronary CTO lesions.

## Materials and Methods

### Patient Population

**Figure 1:**
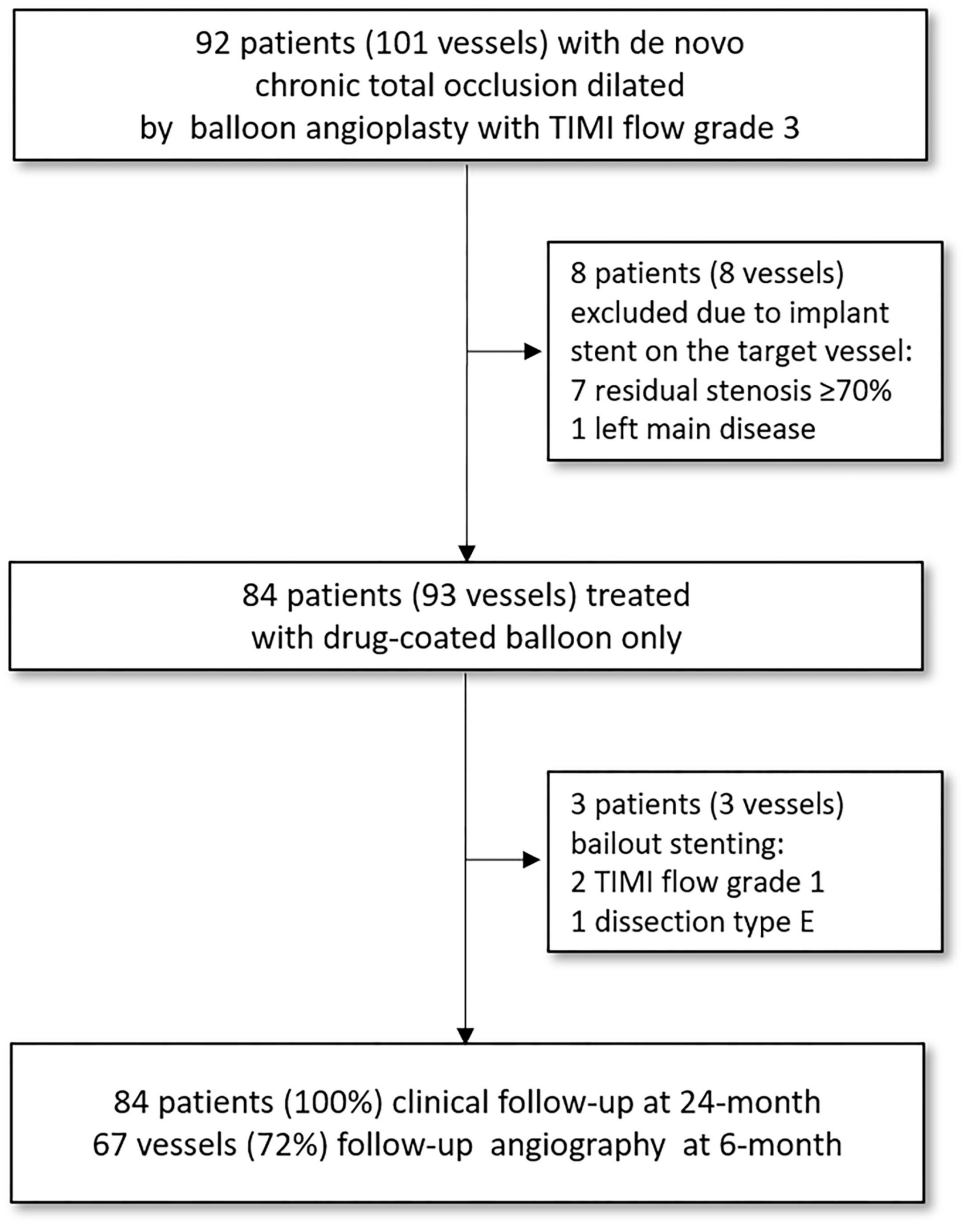
Flow chart of the study. The study population consisted of 93 vessels with *de novo* coronary CTO lesions that had TIMI flow grade 3 and <50% of residual stenosis following pre-dilatation balloon angioplasty and were successfully treated with DCB without requiring stent implantation. All patients were followed clinically, and angiographic follow-up was performed in 72% of the vessels under study. CTO, chronic total occlusion; TIMI, thrombolysis in myocardial infarction.

This retrospective observational study was conducted at two centers (Queen Elizabeth Hospital II and Ulsan Medical Center) experienced in treating patients with *de novo* CTOs with DCB (SeQuent® Please; B. Bruan Melsungen AG, Berlin, Germany or IN.PACT™ Admiral™; Medtronic, Minneapolis, USA) treatment (Clinical Trial Registration Information; Impact of Drug-coated Balloon Treatment in *De Novo* Coronary Lesion; NCT04619277). In this study, 101 vessels (92 patients who have consented) with *de novo* CTO lesions dilated using balloon angioplasty with thrombolysis in myocardial infarction (TIMI) flow grade 3 were included. Among them, all patients with successful PCI (defined as TIMI flow grade 3 and residual stenosis of ≤ 50%) for *de novo* CTO lesions performed using only DCB were eligible for inclusion. Patients treated with DCB for CTO due to occlusive ISR, stenting of the vessel during the index procedure, or unstable hemodynamic conditions at presentation were excluded. All patients were followed up clinically, and angiographic follow-up was performed in 72% of the vessels (Figure 1). The study protocol was approved by the institutional review board at each participating center, and all patients provided written informed consent to participate at the time of enrollment.

### Procedure

All interventions were performed using the antegrade approach for recanalization. The intervention was performed according to the International DCB Consensus and the Asia-Pacific Consensus on DCB PCI (11, 12). Specifically, an optimal-sized pre-dilation balloon, including a scoring balloon, was mandatorily used at the recommended balloon-to-vessel ratio of 0.8–1.0. After pre-dilation balloon angioplasty, stenting was deferred in all types of dissections (A to E) (13), provided there was TIMI flow grade 3. In cases of flow-limiting dissection after pre-dilation balloon angioplasty (TIMI flow grade of **<**3), more than 50% of residual stenosis, and left main disease, PCI with stent insertion was recommended without using a DCB. The DCB was inflated for at least 60 s with its nominal pressure. After DCB use, a final assessment was undertaken at least 5 min after injection of intracoronary vasodilators, such as nitroglycerin, to catch early vessel closure. In this event, bailout stenting was considered. A bailout glycoprotein IIb/IIIa receptor inhibitor strategy was allowed in cases of high thrombus burden (14). After PCI, even though the duration of the prescribed dual-antiplatelet therapy was left to the attending doctors' discretion, almost all patients received dual-antiplatelet therapy for at least 1 month with life-long continuation of aspirin once daily afterward.

### Definitions

Chronic total occlusions are defined as completely occluded coronary arteries without antegrade coronary flow with a duration of more than 3 months (15). Angiographic success was defined as a final residual stenosis of ≤ 50% on quantitative coronary angiography (QCA), with TIMI flow grade 3 after DCB treatment. Procedural success was defined as angiographic success without the occurrence of in-hospital adverse cardiac events (defined as any occurrence of cardiac death, non-fatal myocardial infarction [MI], target vessel revascularization [TVR], or target vessel thrombosis). Binary restenosis was defined as stenosis of at least 50% of the luminal diameter at the angiographic follow-up.

### Endpoints

The study endpoint was cumulative major adverse cardiac events (MACE), a composite of cardiac death, non-fatal MI, TVR, and target vessel thrombosis, during the 2-year follow-up. The angiographic endpoint was late lumen loss (LLL) at the 6-month scheduled follow-up angiography.

### Statistical Analysis

Analyses were performed on a per-patient basis for clinical characteristics and primary outcome (and its individual components) and a per-vessel basis for vessel-related parameters and vessel-level clinical outcomes. Categorical variables are presented as numbers with relative frequencies (percentages) and continuous variables as means with standard deviations or medians with interquartile ranges (IQRs; first and third quartiles), according to their distributions determined using the Kolmogorov–Smirnov test. For demographic characteristics, continuous data were summarized as descriptive statistics (i.e., number of subjects, means, and standard deviations) and categorical data as frequencies and fractions. To compare two groups, the independent two-sample *t*-test or Wilcoxon rank-sum test was used. If necessary, the chi-square test or Fisher's exact test was performed to compare the two groups. All statistical analyses were performed at a two-sided significance level of 0.05 using Statistical Package for the Social Sciences (version 21.0; IBM Corporation, Armonk, NY, USA), NCSS (NCSS LLC, East Kaysville, UT, USA), and R (version 3.6.3; R Foundation for Statistical Computing, Vienna, Austria).

## Results

The study population consisted of 84 patients with *de novo* coronary CTO lesions (93 vessels) who had TIMI flow grade 3 and **<** 50% of residual stenosis following pre-dilatation balloon angioplasty, and were successfully treated with DCB without requiring stent implantation. The median follow-up duration was 720 days (IQR, 406–1,268 days) after the index procedure. Angiographic follow-up was not compulsory, but encouraged, and was performed in 72% of the vessels (Figure 1). Bailout stenting was required in three patients (3 vessels) after DCB treatment (due to TIMI flow grade 1 in 2 vessels and type E dissection in 1 vessel; all these lesions were treated with new-generation DES). Overall, the angiographic success rate was 95.7%, and no in-hospital adverse cardiac events occurred (**Table 4**).

The baseline clinical characteristics of the included subjects are presented in Table 1. The mean age was 56 years, and most patients were male (85.7%), with only over a third having diabetes mellitus. The mean left ventricular ejection fraction was 50.4 ± 12.9% (the ejection fraction in 9.5% of the patients was ≦35%). Clinical presentations were chronic stable angina (61.9%) and acute coronary syndrome (38.1%).

**Table 1:** Baseline patient characteristics.

	***n* = 84 patients**
Age,years	56.1 ± 9.9
Male	72 (85.7)
Hypertension	49 (58.3)
Hypercholesterolemia	40 (47.6)
Diabetes	32 (38.1)
Current smoker	16 (19.0)
Previous myocardial infarction	21 (25.0)
Previous percutaneous intervention	21 (25.0)
Previous coronary artery bypass grafting	0
Previous stroke	6 (7.1)
Family history of coronary artery disease	26 (31.0)
Hemodialysis	1 (1.2)
Ejection fraction, %	50.4 ± 12.9
Clinical presentation	
Chronic stable angina	52 (61.9)
Acute coronary syndrome	32 (38.1)

*Values are mean ± SD or number (percentage)*.

**Figure 2:**
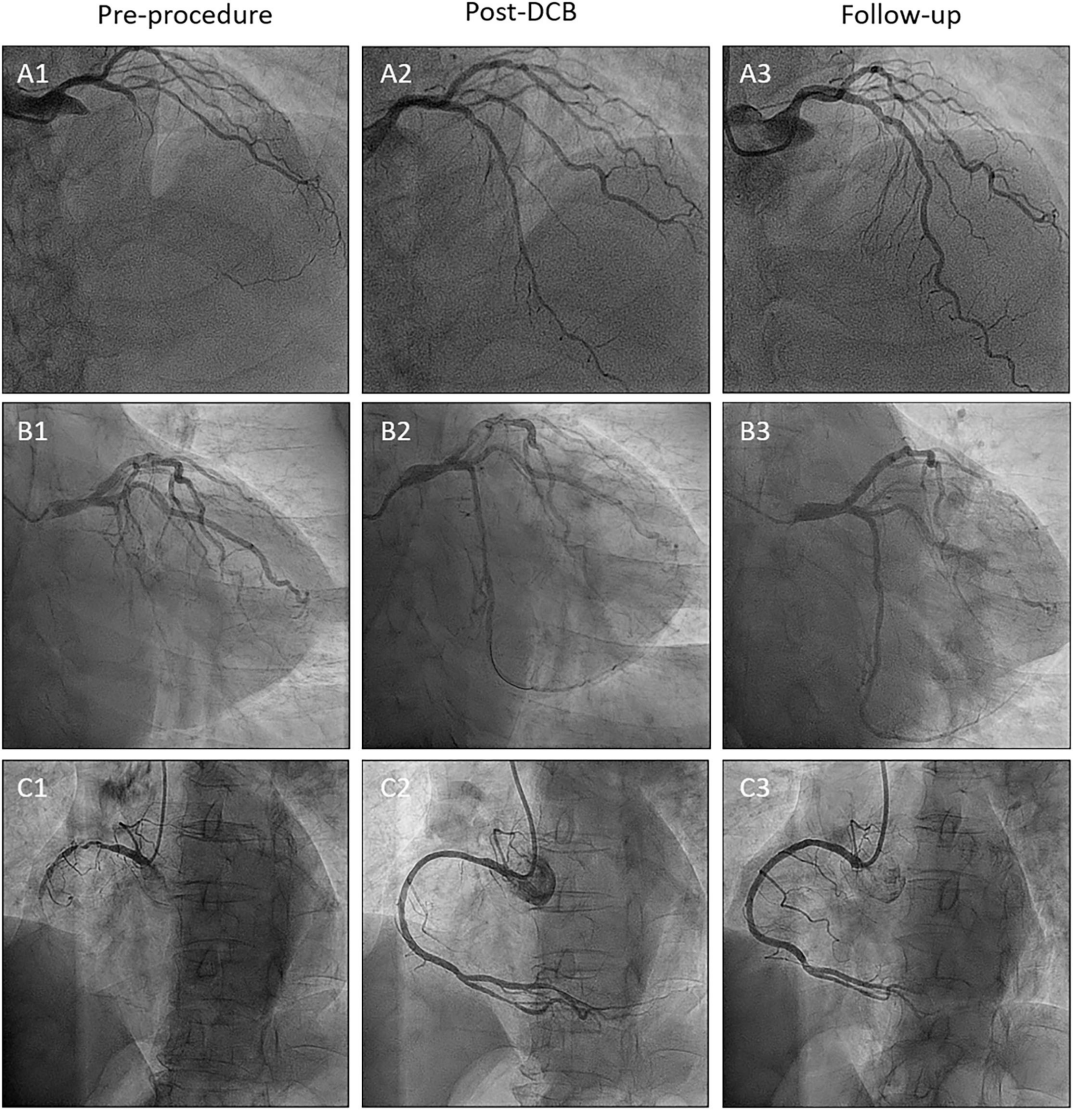
Representative CTO cases. **(A)** Left anterior descending artery lesion. **(B)** Left circumflex artery lesion. **(C)** Right coronary artery lesion. Number 1; Preprocedure. Number 2; Post-DCB treatment. Number 3; Follow-up. CTO, chronic total occlusion.

Table 2 summarizes the angiographic and procedural data. Most patients were treated *via* radial access, and the median SYNergy between PCI with TAXUS and Cardiac Surgery (SYNTAX) score was 21.5 (IQR; 15.5–28.0; 53.8% <23 points, 28% 23–32 points, and 18.3% >32 points). In 48.4% of cases, the left anterior descending artery was treated, and a scoring balloon was used to treat a fifth of the vessels. The mean pre-dilation balloon diameter was 2.4 ± 0.5 mm and the mean DCB diameter was 2.7 ± 0.4 mm. SeQuent® Please DCB was used in most cases (80.6%). The mean number of used DCBs was 1.5 ± 0.6 and the DCB to reference vessel ratio was 1.0 ± 0.2. The mean DCB length was 42.3 ± 17.1 mm. The mean inflation time was 72.0 ± 29.7 s. After DCB treatment, 73.1% of the vessels had dissections (23.7%, type A; 43.0%, type B; 4.3%, type C; 1.1%, type D; and 1.1%, type E). Chronic stable angina (66.2%) and left anterior descending artery (51.5%) were more frequent in the dissection group. One case of type E dissection underwent bailout stenting using a DES. Almost all dissections disappeared (93.6%) at the follow-up coronary angiography. Representative images of the impact of DCB treatment on *de novo* CTO are presented in Figure 2.

**Table 2:** Angiographic and procedural characteristics.

	***n* = 93 vessels**
Radial artery access	82 (88.2)
Syntax score, median (IQR)	21.5 (15.5–28.0)
J-CTO score, mm	1.4 ± 0.6
Number of diseased vessels	1.8 ± 0.8
Targeted vessel	
Left anterior descending artery	45 (48.4)
Left circumflex artery	24 (25.8)
Right coronary artery	24 (25.8)
Scoring balloon used	19 (20.4)
Pre-dilation balloon diameter, mm	2.4 ± 0.5
DCB type	
SeQuent please family	75 (80.6)
IN.PACT Falcon	18 (19.4)
Number of DCB used	1.5 ± 0.6
DCB diameter, mm	2.7 ± 0.4
DCB to reference vessel ratio	1.0 ± 0.2
DCB length, mm	42.3 ± 17.1
DCB inflation time, second	72.0 ± 29.7
Dissection type after DCB	
None	25 (26.9)
A	22 (23.7)
B	40 (43.0)
C	4 (4.3)
D	1 (1.1)
E	1 (1.1)
Bail-out stenting	3 (3.2)
Dissection at follow-up	*N* = 67
None	64 (95.5)
A	0
B	3 (4.5)
C	0

*Values are mean ± SD or number (percentage). IQR, interquartile range; J-CTO, Japan chronic total occlusion; DCB, drug-coated balloon*.

### Clinical Outcomes

Table 3 summarizes the procedural complications and clinical outcomes. No procedural and in-hospital complications, such as perforation, pericardiocentesis, emergency bypass surgery, or stroke, were observed in this registry. MACE occurred in 8.3% of the patients 1 year after the procedure, including cardiac death (1.2%) and TVR (7.1%). MACE occurred in 16.7% of the patients 2 years after the procedure, including cardiac death (2.4%), non-fatal MI (3.6%), and TVR (13.1%); however, no target vessel thrombosis occurred. Among the two cases of cardiac death, one died 4 months after the follow-up due to decompensated heart failure secondary to pneumonia, and the other died out-of-hospital 9 months after the follow-up due to a heart attack. Of the TVR cases, six were treated with DES, one underwent coronary artery bypass graft surgery, and three underwent repeat DCB. The rate of new vessel revascularization was 10.7%.

**Table 3:** Procedural complications and clinical outcomes.

	***n* = 84 patients**
Procedural and in-hospital complication	
Perforation	0
Pericardiocentesis	0
Emergency coronary artery bypass grafting	0
Stroke	0
Clinical outcomes at 1 year	
Major adverse cardiac events	7 (8.3)
All death	1 (1.2)
Cardiac death	1 (1.2)
Non-fatal myocardial infarction	0
Target lesion revascularization	6 (7.1)
Target vessel revascularization	6 (7.1)
New vessel revascularization	9 (10.7)
Target vessel thrombosis	0
Stroke	2 (2.4)
Clinical outcomes at 2 years, day, median (IQR)	720 (406–1268)
Major adverse cardiac events	14 (16.7)
All death	2 (2.4)
Cardiac death	2 (2.4)
Non-fatal myocardial infarction	3 (3.6)
Target lesion revascularization	11 (13.1)
Target vessel revascularization	11 (13.1)
New vessel revascularization	9 (10.7)
Target vessel thrombosis	0
Stroke	2 (2.4)

*Values are mean ± SD or number (percentage)*.

*IQR, interquartile range; MACE, major adverse cardiac events; a composite of cardiac death, non-fatal myocardial infarction, target vessel revascularization, and target vessel thrombosis*.

### Angiographic Outcomes

After the index procedure, 61 (72.6%) patients returned for scheduled follow-up angiography at a median duration of 186 days (IQR, 134–291 days), ensuring that serial QCA data were available for 67 vessels. The angiographic outcomes are presented in Table 4, and representative cases of each coronary artery are shown in Figure 2. Due to the nature of the CTO, the baseline QCA data required to calculate the LLL were only available after DCB treatment. The mean reference vessel diameter (RVD) after DCB treatment was 2.3 ± 0.5 mm, while the mean residual diameter stenosis was 30.6 ± 9.3%. The acute lumen gain was 1.6 ± 0.4 mm. Binary restenosis occurred in 14.9% of the treated vessels, among which two (3.0%) lesions were totally re-occluded at the follow-up angiography. LLL was 0.03 ± 0.53 mm, with 37 (55.2%) lesions having LLL values below 0 due to an increase in vessel size.

**Table 4:** Quantitative coronary angiography and late luminal loss.

	***n* = 93 vessels**
Post-DCB treatment	
Reference vessel diameter, mm	2.3 ± 0.5
Lesion length, mm	42.4 ± 17.0
Minimal lumen diameter, mm	1.6 ± 0.4
Diameter stenosis, %	30.6 ± 9.3
Acute lumen gain, mm	1.6 ± 0.4
Follow-up	*n* = 67 vessels
Reference vessel diameter, mm	2.5 ± 0.7
Lesion length, mm	43.7 ± 16.8
Minimal lumen diameter, mm	1.6 ± 0.6
Diameter stenosis, %	37.8 ± 17.3
Late lumen loss, mm	0.03 ± 0.53
Binary restenosis	10/67 (14.9)
Scheduled angiography follow-up duration, day, median (IQR)	186 (134–291)

*Values are mean ± SD*.

*DCB, drug-coated balloon; IQR, interquartile range*.

**Figure 3:**
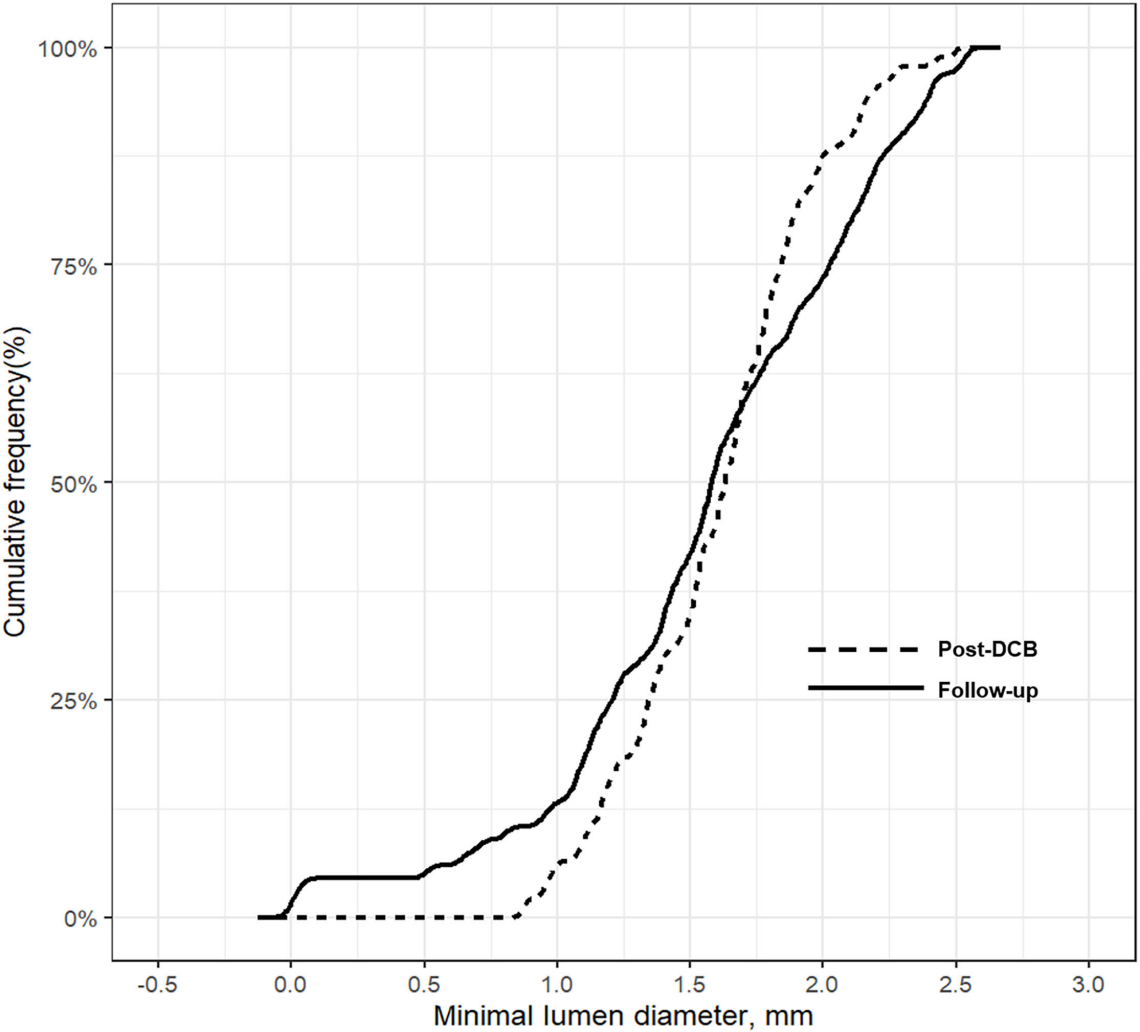
Cumulative frequency distribution of MLD. MLD, minimal lumen diameter.

No significant changes in the minimum lumen diameter (MLD) were observed between the index procedure and follow-up angiography (post-DCB vs. follow-up, 1.6 ± 0.4 mm vs. 1.6 ± 0.6 mm; *p* = 0.675 (Table 4 and Supplementary Table 1); however, the RVD was significantly larger at follow-up than after DCB treatment (post-DCB vs. follow-up, 2.3 ± 0.5 mm vs. 2.5 ± 0.7 mm; *p* = 0.033); hence, the diameter stenosis significantly increased at follow-up (post-DCB vs. follow-up, 31.5 ± 9.0% vs. 37.8 ± 17.3%; *p* = 0.005). Figure 3 presents the cumulative frequency distributions of MLD.

## Discussion

In this study, we investigated the feasibility and clinical outcomes of DCB only treatment for *de novo* coronary CTO lesions. The main findings were as follows; first, after successful balloon angioplasty of *de novo* CTO lesions, DCB only treatment is feasible and effective; second, the rate of MACE, the primary endpoint, was 16.7%, and was driven primarily by TVR without target vessel thrombosis during a median follow-up of 720 days; third, LLL, the angiographic outcome, was minimal (0.03 ± 0.53 mm), confirming the efficacy of DCB in inhibiting neointimal hyperplasia.

Although DES has significantly reduced the incidence of restenosis and clinical events compared with bare-metal stents, risks associated with their use remain, such as neoatherosclerosis due to their pro-inflammatory effect, and ST due to delayed neointimal coverage (16, 17). Using first-generation DESs, ST accrued at a rate of 0.6% per year (18), while rates fell to 0.3% per year using second-generation DESs. Nevertheless, this risk persists for at least 5 years after stent insertion (19). In a recent large-scale, individual patient-level pooled study (*n* = 25,032), very-late ST occurred between 1 and 5 years after PCI at a rate of 0.4% per year with all stent types, without an evident plateau (20). In contrast to DES, DCB can reduce this risk, while eluting drugs for a short time can effectively prevent neointimal hyperplasia in the absence of foreign substances, as shown in several DCB experiments with long-term follow-up (21, 22). In the Swedish Coronary Angiography and Angioplasty Registry, the rate of target lesion thrombosis in *de novo* lesions in small coronary vessels undergoing PCI was 0.6% at 6 months, without events occurring between 6 months and the final follow-up at 3 years (22). In the BASKET-SMALL 2 trial, a multicenter, open-label, randomized non-inferiority trial for small native coronary artery disease (n = 758), probable or definite ST was lower using DCBs (DCB vs. DES; 0.8% vs. 1.1%; hazard ratio [HR], 0.73 [95% confidence interval [CI], 0.16–3.26]) (21). Recently, they demonstrated that no significant difference in the rate of MACE (15% in both groups) has been shown between the DCB and DES groups (HR, 0.99; 95% CI, 0.68–1.45; *p* = 0.95) for 3 years. The rates of probable or definite ST (Kaplan–Meier estimate, 1% vs. 2%; HR, 0.33; 95% CI, 0.07–1.64; *p* = 0.18) and major bleeding (Kaplan–Meier estimate, 2% vs. 4%; HR, 0.43; 95% CI, 0.17–1.13; *p* = 0.088) were numerically lower in the DCB group than in the DES group.

In this study, there were no cases of target vessel thrombosis during a median follow-up of 720 days, despite a similar lesion severity compared with previous CTO studies (20, 23). In the DECISION-CTO trial (24), the SYNTAX score was 20.8 ± 9.2 and the mean stent length was 41.1 ± 25.9 mm. In comparison, the median SYNTAX score in this study was 21.5 (IQR, 15.5–28.0), and the mean lesion length was 42.3 ± 17.1 mm, which was not significantly different from that of the CTO lesions treated with stents in previous studies (25, 26). Nevertheless, the MACE results in this study were similar to those detected by the DECISION-CTO trial, which used second-generation DES in 82.6% of patients. In the DECISION-CTO trial, the rate of cardiac death, MI, and TVR were 1.9%, 11.3%, and 7.9%, respectively; this study showed comparable event rates (i.e., 2.4%, 3.6%, and 13.1%, respectively). In the DECISION-CTO trial, the rate of definite ST was 0.2% during a median follow-up of 4 years, while the rate of definite or probable ST was 0.4% in the EURO-CTO trial at the 12-month follow-up (25).

The high risk of restenosis and ST in patients with CTO remains a challenging problem. One of the mechanisms for these clinical events is that stents in newly opened CTOs are commonly undersized because the vessel does not grow immediately after recanalization (27). Vessels distal to a CTO are narrow because of the absence of blood flow for a long time. After balloon angioplasty, the antegrade flow increases and the vessels become more expansive; however, this may take several weeks or months. Therefore, immediately after balloon angioplasty of a CTO, underestimating the actual vessel size is easy, thus increasing the risk of stent undersizing and subsequent risks of restenosis, late stent malapposition, and ST. Moreover, the metallic cage can inhibit positive remodeling, leaving a small luminal size after vessel recovery. However, after DCB treatment, vessels may return to their original sizes over time, without fixing the size of the vessel using a foreign body, such as a metal stent, which is one of the most appealing and important advantages of using DCB in treating CTO lesions. These findings show the feasibility of DCB as a treatment for CTO lesions, especially in patients with poor medication compliance (28) or those at a high bleeding risk (29, 30). Additionally, the hybrid approach, where a DES is implanted at the proximal segment of a CTO lesion and the distal lesion is treated with a DCB, may reduce the risk of stent-related events by reducing the stent burden. Only a few studies have investigated the impact of DCB only treatment on *de novo* CTO lesions (31, 32). According to a recent study, DCBs were successfully used for two totally occluded lesions in a patient with acute coronary syndrome (7). After 3 months, follow-up angiography confirmed adequate patency of the DCB-treated lesions, without symptoms until 13 months. Recently, Koln et al. have shown that the use of DCB is a feasible and well-tolerated treatment that provided promising results from pre-dilation (9). They have shown that the positive late lumen gain in 67.6% of the patients was due to increased vessel size following DCB treatment. This is comparable to our data, which confirmed positive late lumen gain in 55.2% of the patients under study.

The most compelling advantage of this study is that DCB treatment without stenting might be a safe and effective modality for treating *de novo* CTO lesions if the result after pre-dilation is good with TIMI flow grade 3. However, this study has several limitations. First, the population is limited and comes from only two expert centers with this type of PCI. Thus, the findings of this study may not be reproducible everywhere without an adequate learning curve. Second, this study did not target all-comer CTO lesions, but consisted only of patients who were successfully re-perfused by pre-dilation and received DCB treatment. Therefore, the results of this study should be interpreted with caution. Prospective and randomized large-scale studies are needed to clarify the efficacy of DCB treatment in *de novo* CTO lesions and compare the efficacy with that of DES implantation.

## Conclusions

If the revascularization result of balloon angioplasty is good, the clinical outcomes of DCB only treatment for *de novo* CTO lesions are encouraging at the 2-year follow-up, with a low rate of hard endpoints and acceptable MACE rates.

## Data Availability Statement

The original contributions presented in the study are included in the article/Supplementary Materials, further inquiries can be directed to the corresponding author/s.

## Ethics Statement

The studies involving human participants were reviewed and approved by Queen Elizabeth Hospital II and Ulsan Medical Center. The patients/participants provided their written informed consent to participate in this study. Written informed consent was obtained from the individual(s) for the publication of any potentially identifiable images or data included in this article.

## Author Contributions

E-SS and HL contributed substantially to the design of this study. EJ and E-SS provided the first draft of the manuscript. EJ, E-VT, and YB performed the data and statistical analyses. All coauthors participated in interpreting the data and critically revised the manuscript. The authors confirm that the manuscript has been blinded to follow the double-blind peer review model. All authors have approved the final version of the manuscript to be submitted. E-SS and HL had full access to the database and take responsibility for the integrity of the data and the data analyses. All authors contributed to the article and approved the submitted version.

## Conflict of Interest

The authors declare that the research was conducted in the absence of any commercial or financial relationships that could be construed as a potential conflict of interest.

## Publisher's Note

All claims expressed in this article are solely those of the authors and do not necessarily represent those of their affiliated organizations, or those of the publisher, the editors and the reviewers. Any product that may be evaluated in this article, or claim that may be made by its manufacturer, is not guaranteed or endorsed by the publisher.

## Abbreviations

CTO: chronic total occlusion
DCB: drug-coated balloon
DES: drug-eluting stent
ISR: in-stent restenosis
IQR: interquartile range
LLL: late lumen loss
MACE: major adverse cardiac events
MI: myocardial infarction
PCI: percutaneous coronary intervention
ST: stent thrombosis
TIMI: thrombolysis in myocardial infarction
TLR: target lesion revascularization
TVR: target vessel revascularization.
