# Supplementary material for: Clinical Outcomes of Drug-Coated Balloon Treatment After Successful Revascularization of de novo Chronic Total Occlusions
Source: Front Cardiovasc Med. 2022 Apr 13;9:821380. doi: 10.3389/fcvm.2022.821380 (PMC9043519; doi:10.3389/fcvm.2022.821380)
Supplement: Supplementary file 1 [file Table_1.DOCX]

**SUPPLEMENTARY TABLE 1┃**Serial change of quantitative coronary angiography

| n = 67 vessels | Post-DCB | Follow-up | *p* value |
| --- | --- | --- | --- |
| Reference vessel diameter, mm | 2.3 ± 0.5 | 2.5 ± 0.7 | **0.033** |
| Lesion length, mm | 43.8 ± 16.8 | 43.7 ± 16.8 | 0..501 |
| Minimal lumen diameter, mm | 1.6 ± 0.4 | 1.6 ± 0.6 | 0.675 |
| Diameter stenosis, % | 31.5 ± 9.0 | 37.8 ± 17.3 | **0.005** |

Values are mean ± SD.

DCB = drug-coated balloon
